# Hematological and performance adaptations to altitude training (2,320 m) in elite middle-distance and distance swimmers 

**DOI:** 10.3389/fphys.2024.1474479

**Published:** 2024-09-23

**Authors:** Iñigo Mujika, Nicolas Bourdillon, Irina Zelenkova, Frédéric Vergnoux, Grégoire P. Millet

**Affiliations:** ^1^ Department of Physiology, Faculty of Medicine and Nursing, University of the Basque Country, Leioa, Spain; ^2^ Exercise Science Laboratory, School of Kinesiology, Faculty of Medicine, Universidad Finis Terrae, Santiago, Chile; ^3^ Institute of Sport Sciences, University of Lausanne, Lausanne, Switzerland; ^4^ Growth, Exercise, Nutrition and Development (GENUD) Research Group, Faculty of Health and Sport Sciences, University of Zaragoza, Zaragoza, Spain; ^5^ Cercle des Nageurs d’Antibes, Antibes, France

**Keywords:** hemoglobin mass, blood volume, hypoxia, swimming, body composition

## Abstract

**Purpose:**

Elite swimmers often schedule altitude training camps ahead of major events in an attempt to maximize performance. However, the relationships between altitude-induced hematological changes, markers of training adaptation, and performance changes in such context are unclear. This study assessed hematological status, markers of daily adaptation, and swimming performance in elite middle-distance and distance swimmers during a 22-day altitude training camp at 2,320 m, 2 weeks prior to World Championship qualification competition.

**Methods:**

Venous blood was obtained and total hemoglobin mass (tHb_mass_) measured (CO rebreathing) in 7 elite swimmers (4 females, 3 males) 8 days before and on day 22 of the altitude camp. Resting heart rate, peripheral oxygen saturation, urinary specific gravity, body mass, fatigue and self-reported sleep duration and quality were monitored daily during the altitude camp. Swimming performance was assessed through a standardized set (6 sets of 4 maximal repetitions of 100 m front crawl) on days 3, 10 and 17 of the camp, and at sea level competitions (200 m–1,500 m) immediately after the camp, and 2 weeks later.

**Results:**

tHb_mass_ (+5.6 ± 3.3%; range: 2.1%–11.0%; *p* < 0.05), red blood cell count, hemoglobin concentration, hematocrit increased at the end of the training camp (*p* < 0.05). Performance at altitude improved throughout the camp (+1.4 ± 0.4%; range: 0.7%–2.5%; *p* < 0.05). No significant relationship was noted between hematological changes, the change in altitude performance and any of the monitored daily markers of adaptation during the camp. Compared to the swimmers’ previous personal best, competition performances did not improve immediately (2.5% ± 1.9% slower times) and 2 weeks after altitude (1.2% ± 1.4% slower times).

**Conclusion:**

The 22-day altitude training camp at 2,320 m was beneficial for elite swimmers’ tHb_mass_, hematological status and performance at altitude, but these benefits did not clearly translate into enhanced sea level performance immediately after or 2 weeks later. The present study confirms the large inter-individual variability in hematological responses to altitude training, and that the improvement in performance at altitude and sea level may depend on factors other than the increase in tHb_mass_ alone.

## 1 Introduction

Numerous national swimming federations schedule altitude training camps ahead of major competitions in an attempt to maximize swimmers’ performance. These camps typically last 2–4 weeks, are often held at altitudes ranging between ∼1,800 and ∼2,500 m, and are mainly planned to induce hematological adaptations (e.g., an increase in total hemoglobin mass [tHb_mass_]) and subsequently enhance sea level performance (Rodríguez et al., 2015; Pla et al., 2020). Despite the technical difficulty of tHb_mass_ assessment (Rodríguez et al., 2015; Schmidt and Prommer, 2005) and difficulties inherent to accessing data on elite athletes, previous research demonstrated that such altitude training camps can induce tHb_mass_ enhancements in elite junior (Friedmann et al., 2005) and senior swimmers (Wachsmuth et al., 2013; Bonne et al., 2014; Gough et al., 2012; Robertson et al., 2010; Astridge et al., 2024).

Available studies analyzing altitude-induced increments in swimmers’ tHb_mass_ and subsequent sea level performance, however, failed to demonstrate a clear impact of the former on the latter (Rodríguez et al., 2015; Wachsmuth et al., 2013; Bonne et al., 2014; Gough et al., 2012). For instance, Robertson et al. (Robertson et al., 2010) found only a moderate correlation between altitude-induced increases in tHb_mass_ and a 2,000 m time trial performance, and an unclear correlation with swimming velocity at the lactate threshold. Recently, Mujika et al. (Mujika et al., 2023) reported that tHb_mass_ and blood volume did not correlate with sea level competition performance, and that peak performances were not attained in temporal concomitance with peak tHb_mass_ values. Such findings suggest that beyond hematological adaptations, cardiovascular factors associated with the supine posture during swimming (Wachsmuth et al., 2013), non-hematological changes incurred during altitude training (e.g., improved buffering capacity) (Gore et al., 2007), biomechanical and coordinative factors affecting the energy cost of swimming (Seifet et al., 2014), as well as optimal periodization and peaking strategies (Gough et al., 2012; Mujika et al., 2019) may be stronger determinants of post-altitude swimming performance.

Daily monitoring of athletes’ adaptations and acclimatization process is paramount during altitude camps, since there is a large inter-individual variability in the responses to altitude exposure (Hauser et al., 2017) due to several potential confounding factors, such as injuries or illnesses (Gough et al., 2013), initial level of ferritin (Bergeron et al., 2012), inflammation or hydration status. The focus on the effectiveness of altitude training in elite endurance athletes (Millet and Brocherie, 2020) has therefore recently been placed on the factors that may blunt the positive altitude-induced adaptations (e.g., inadequate planning, periodization, programming, and monitoring) (Mujika et al., 2019). For instance, Astridge et al. (2024) did not observe improved sea level performances neither immediately nor 3 weeks after a 3-week altitude camp, in spite of persistently enhanced values of tHb_mass_. These authors suggested the need for characterization of the highly individual relationship between hypoxic dose, training load periodization, hematological status and sea level performance.

The present study assessed hematological status, markers of daily adaptation, and swimming performance in a group of elite middle-distance and distance swimmers during and after a 22-day training camp at an altitude of 2,320 m, 2 weeks prior to the 2019 FINA World Championship qualifiers.

## 2 Methods

### 2.1 Participants

Seven elite swimmers (6 Tier 4, 1 Tier 5 (McKay et al., 2022); 4 females, age 22.5 ± 4.4 years, height 168.3 ± 3.8 cm, mass 62.8 ± 2.6; 3 males, 21.0 ± 1.4 years, 177.0 ± 4.0 cm, 69.4 ± 2.4) participated in a 22-day altitude training camp. All swimmers were members of the Spanish pool or open water national teams, specialized in events ranging from 400 to 1,500 m and/or 10 km open water, had extensive previous altitude training experience, and participated in major international competitions. The sample included one Olympic and World champion, five participants at the 2019 FINA World Championship, and four Tokyo 2020 Olympians.

### 2.2 Experimental design

**FIGURE 1:**
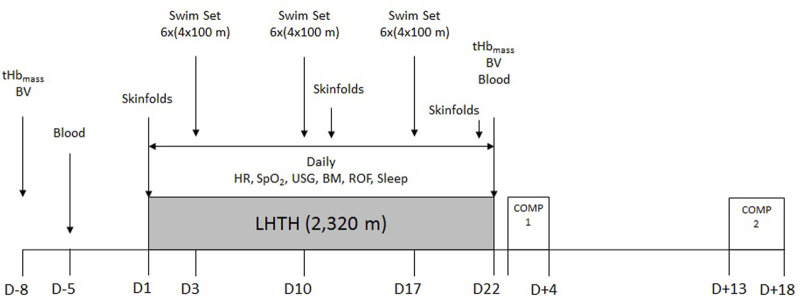
Timeline of the experimental design and procedures. tHb_mass_: total hemoglobin mass; BV: blood volume; Blood: venous blood sampling; HR: resting heart rate; SpO_2_: resting peripheral oxygen saturation; USG: urinary specific gravity; BM: body mass; ROF: rating of fatigue; Sleep: self-reported sleep duration and quality; LHTH: live-high, train-high altitude training camp; COMP: competition.

A single-group research design was implemented, as the elite level of the participants precluded the implementation of a control group of similar caliber. The altitude camp was held at the Sierra Nevada High Performance Center (2,320 m) between February and March 2019, finishing 2 weeks before the qualification competition for the 2019 FINA World Championship (Figure 1). Swimming volume during the camp was 316.3 ± 35.6 km and ∼25 h of dryland training. A typical training week consisted of 45 min of dryland aerobic training before breakfast, three times per week on alternate days (including stationary cycling, rowing and/or running); daily 2.5-h morning (after breakfast) and afternoon (after lunch and nap) swim sessions (except for full rest on Wednesday afternoon and Sunday morning); 1 h of circuit strength training three times per week (after the morning swim session, before lunch) on alternate days; and 45 min of core stability training three times per week (following the afternoon swim session, before dinner) on alternate days. All athletes ingested a daily morning dose of 100 mg of ferrous glycine sulphate (Ferbisol 100 mg, Aesica Pharmaceuticals GmbH, Monheim, Germany) 2 weeks before, during, and 2 weeks after the altitude camp. All procedures were part of the team’s service provision, which conforms to the Code of Ethics of the World Medical Association (Declaration of Helsinki). Athletes provided informed consent to participate in monitoring procedures associated with team duties, with the understanding that data may be used for research purposes.

### 2.3 Procedures

#### 2.3.1 Total hemoglobin mass and blood volume

tHb_mass_ (g) and blood volume (mL) were measured at sea level 8 days before the start of the camp and on the last day of the camp, using an optimized carbon monoxide (CO)-rebreathing method (Schmidt and Prommer, 2005). The CO dose was 1.0 mL/kg body mass for male and 0.8 mL/kg for female swimmers. The rebreathing procedure was performed for 2 min through a glass spirometer (Blood tec GmbH, Bayreuth, Germany). Fingertip capillary blood samples (200 μL) were obtained immediately before the test, and six and 8 min following CO inhalation for determination of %HbCO (ABL80 FLEX CO-OX analyzer, Radiometer, Copenhagen, Denmark). Hemoglobin concentration (g/dL) and hematocrit (%) were determined in capillary blood (Hemo Control, EKF Diagnostics, Cardiff, United Kingdom). All measurements of tHb_mass_ were performed by the same experienced researcher using the same equipment, with a typical error of 6.0 g in duplicated measurements with 24 h gap between measurements.

#### 2.3.2 Blood analyses

Five days before the camp and on the last day of the camp fasted forearm venous blood samples were collected to perform hematological analyses through standard procedures in an accredited clinical analysis laboratory. Briefly, the swimmers reported to the same clinical analysis laboratory after overnight fasting. Blood was collected by venepuncture from an antecubital vein after 20 min of seated rest. The samples were analyzed for full hematology, including red cell count and iron status (serum iron, total iron-binding capacity, transferrin, saturation index and ferritin).

#### 2.3.3 Daily assessments

Daily assessments of swimmers’ adaptations included resting heart rate (HR) and peripheral oxygen saturation upon waking (SpO_2_, Beurer PO 30, Ulm, Germany); morning mid-stream urinary specific gravity (USG, UG-α Digital Refractometer, Atago Co., Tokyo, Japan); body mass monitored after voiding (Seca 877, Hamburg, Germany); Rating of Fatigue (Micklewright et al., 2017); self-reported sleep hours; subjective sleep quality using a Likert scale (5 = very good; 4 = good; 3 = average; 2 = bad; 1 = very bad) (Saw et al., 2018).

#### 2.3.4 Skinfolds

Skinfolds were measured by the same accredited anthropometrist using a standard protocol for the sum of 7 sites (triceps, biceps, subscapular, supraspinal, abdominal, front thigh, medial calf; Holtain, Crymych, United Kingdom) at the start of the camp, mid-camp, and on the second last day of the camp.

#### 2.3.5 Performance

The swimmers performed a standardized performance set on days 3, 10 and 17 of the camp, consisting of 6 sets of 4 maximal repetitions of 100 m front crawl, with 75 s of passive recovery between repetitions and a 400-m recovery swim between sets. Each 100 m repetition was manually timed by the same experienced coach. Blood lactate concentration was measured from a 5 μL ear lobe capillary blood sample within the initial seconds of recovery after each swim set (Lactate Pro 2, Arkray Inc., Shiga, Japan). The day after the camp, 6 of the 7 swimmers participated in a 3-day international competition (Meeting Open Méditerranée, Marseille, France): the 3 male swimmers raced in the 200, 400 and 1,500 m freestyle; the 3 females raced in the 200, 400, 800, 1,500 m freestyle and the 400 m individual medley, but two of them also raced in the 200 m butterfly and the 200 m individual medley. Two weeks after the altitude camp, all 7 swimmers participated in the qualification competition for the 2019 FINA World Championship at sea level (Campeonato de España Open, Sabadell, Spain). All 7 swimmers took part in the 400, 800, 1,500 m freestyle events, but two female swimmers also raced in the 400 m individual medley, one of whom raced also in the 200 m butterfly and 200 m individual medley. When a swimmer swam in the same event more than once (i.e., heats and final), only the best performance was retained and subsequently compared with individual personal best time in that event.

### 2.4 Statistics

Data are presented as mean ± SD. Two-way ANOVA for time (D3-D10-D17) and swim set (Rodríguez et al., 2015; Pla et al., 2020; Schmidt and Prommer, 2005; Friedmann et al., 2005; Wachsmuth et al., 2013; Bonne et al., 2014) were performed for performance and lactate analyses. One-way ANOVA for time (D1-D22) was performed on the parameters recorded daily. Paired t-tests were performed for all the other variables to compare PRE (or D1) and D22. On the one hand Pearson’s R correlation coefficients were sought between changes in performance (D3 to D17) and changes in all the other parameters, and on the other hand between changes in tHb_mass_ and changes in all the other parameters, in raw and normalized values. Data normality and homogeneity were evaluated using the Shapiro–Wilk and Bartlett’s tests, respectively, and significance was set at *p* < 0.05 for all statistical tests. All analyses were performed using MATLAB^®^ (R2019a, MathWorks, Natick, MA, United States).

Performance results showed identical significance in men and women, which were therefore pooled for the statistical analyses, but shown separately on figures for better readability.

## 3 Results

**FIGURE 2:**
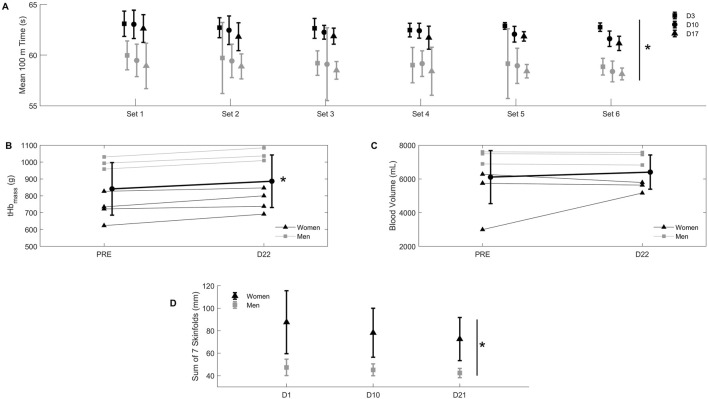
Panel **(A)** mean ± SD 100 m time in seconds during 6 sets of 4 repetitions of 100 m front crawl, with 75 s of passive recovery between repetitions and a 400-m recovery swim between sets, at day 3 (D3, squares), day 10 (D10, circles), and day 17 (D17, triangles) of exposure to 2,320 m of altitude. Black symbols: women; grey symbols: men. *D17 different from D3 in both women and men (*p* < 0.05). Panel **(B)** individual values (thin line) for total hemoglobin mass (tHb_mass_) in grams before (PRE) and on the last day (D22) of exposure to 2,320 m of altitude. Triangles: women; squares: men. Bold line: mean ± SD of all participants. *D22 different from PRE (*p* < 0.05). Panel **(C)** individual values (thin line) for blood volume in milliliters before (PRE) and on the last day (D22) of exposure to 2,320 m. Triangles: women; squares; men. Bold line: mean ± SD of all participants. Panel **(D)** mean ± SD sum of seven skinfolds in millimeters measured at day 1 (D1), day 10 (D10) and day 21 (D21) of exposure to 2,320 m of altitude. Black symbols: women; grey symbols: men. *D21 different from D3 in both women and men (*p* < 0.05).

**FIGURE 3:**
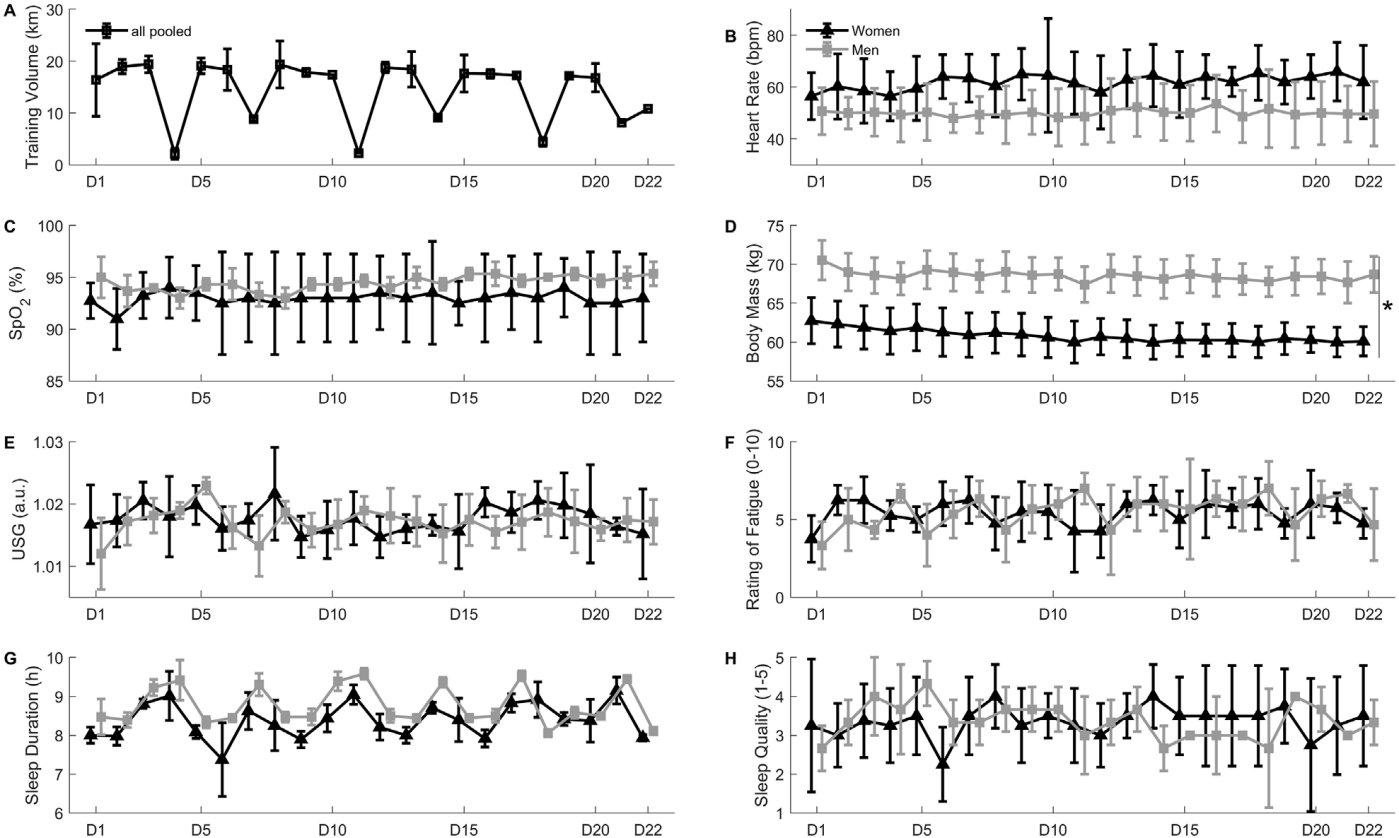
Panel **(A)** Daily training volume in km. Panel **(B)** Resting heart rate in beats per minute. Panel **(C)** Resting peripheral oxygen saturation (SpO_2_) in percent. Panel **(D)** Body mass in kilograms, *D22 different from D1 in both women and men (*p* < 0.05). Panel **(E)** urinary specific gravity (USG), arbitrary units. Panel **(F)** Rating of fatigue on a 0-10 scale. Panel **(G)** sleep duration in hours. Panel **(H)** sleep quality on a 1-5 scale. All values are mean ± SD.

Total hypoxic dose was 528 h or 1,225 km h (Garvican-Lewis et al., 2016). tHb_mass_ increased from PRE to D22, as shown on Figure 2B (+5.6 ± 3.3%; range: 2.1%–11.0%, *p* < 0.05), but blood volume did not change (Figure 2C). Red blood cell count, hemoglobin concentration and hematocrit also increased from PRE to D22 (*p* < 0.05), whilst other hematological parameters also changed (Table 1). Body mass (Figure 3D) and skinfold thickness (Figure 2D) decreased from D1 to D22 (*p* < 0.05).

**TABLE 1:** Parameters measured either before (PRE) and on the last day (D22) of exposure to an altitude of 2,320 m * different from PRE (*p* < 0.05).

	All		Females (n = 4)		Males (n = 3)	
	PRE	D22	PRE	D22	PRE	D22
RBC (10^6^/mL)	4.7 ± 0.4	5.2 ± 0.3*	4.5 ± 0.5	5.2 ± 0.4*	4.8 ± 0.3	5.2 ± 0.3
Hb (g/dL)	14.4 ± 1.0	15.6 ± 0.7*	13.9 ± 0.8	15.5 ± 1.0*	15.0 ± 0.8	15.7 ± 0.2
Hct (%)	42.8 ± 2.4	47.3 ± 1.5*	41.7 ± 2.2	47.4 ± 2.1*	44.4 ± 1.9	47.1 ± 0.2
MCV (fL)	92.5 ± 5.0	90.6 ± 5.1*	92.8 ± 6.2	90.9 ± 6.1*	92.0 ± 4.1	90.3 ± 4.6
MCH (pg)	31.0 ± 2.1	29.8 ± 2.1*	30.9 ± 2.5	29.7 ± 2.7*	31.1 ± 2.0	30.0 ± 1.6
MCHC (g/dL)	33.5 ± 0.7	32.9 ± 0.7*	33.3 ± 0.7	32.6 ± 0.9*	33.8 ± 0.6	33.2 ± 0.5*
RDW (%)	13.1 ± 0.4	13.6 ± 0.4*	13.3 ± 0.4	13.8 ± 0.5*	12.9 ± 0.2	13.3 ± 0.3*
Retic (10^9^/L)	64.0 ± 17.4	60.1 ± 17.1	62.7 ± 17.6	68.5 ± 18.0	65.9 ± 20.8	49 ± 8.2
Retic (%)	1.4 ± 0.4	1.1 ± 0.3	1.4 ± 0.5	1.3 ± 0.4	1.4 ± 0.4	0.9 ± 0.1
Iron (µg/dL)	126.8 ± 47.2	88.1 ± 22.4	128.4 ± 63.4	79.1 ± 26.5	124.7 ± 25.2	100.1 ± 8.6
Ferritin (ng/mL)	88.6 ± 57.8	75.7 ± 38.2	51.3 ± 22.9	48.9 ± 12.4	138.3 ± 52.2	111.3 ± 28.5

RBC: red blood cell count; Hb: hemoglobin concentration; Hct: hematocrit; MCV: mean corpuscular volume; MCH: mean corpuscular hemoglobin; MCHC: mean corpuscular hemoglobin concentration; RDW: red cell distribution width; Retic: reticulocytes.

Although no change in serum iron concentration was found, there was a negative correlation between the change in tHb_mass_ and the change in serum iron from PRE to D22 (R = −0.78, *p* < 0.05), i.e., the swimmers who increased tHb_mass_ the most were those who experienced the greatest decrease in serum iron. No statistically significant relationship was noted between hematological changes, the change in performance at altitude and any of the monitored daily markers of adaptation during the camp.

Swim performance data at altitude are shown in Figure 2A. There was a significant improvement at D17 compared to D3 (+1.4 ± 0.4%; range: 0.7%–2.5%, *p* < 0.05). Blood lactate increased from 4.8 ± 1.8 to 6.3 ± 3.3 mM in sets 1 through 6 on day 3; from 4.2 ± 1.6 to 6.6 ± 4.5 on day 10; and from 4.2 ± 1.2 to 5.8 ± 1.3 mM on day 17 (*p* > 0.05 between testing sessions). Sea level performance failed to improve in comparison with the swimmers’ previous personal best immediately after camp (2.5% ± 1.9% slower times), and only 1 personal best time was achieved. Performance at the qualification competition for the 2019 FINA World Championship 2 weeks after altitude did not improve either compared to the swimmers’ previous personal best (1.2% ± 1.4% slower times), with 21 events slower than previous personal best times, and 4 events faster.

## 4 Discussion

The present study demonstrated a significant increase in tHb_mass_ and swimming performance in elite middle-distance and distance swimmers over a 22-day training camp at an altitude of 2,320 m. However, the observed performance improvement at altitude was not related to the increase in tHb_mass_ or any other hematological variables, and did not translate into enhanced sea level competition performance immediately after, nor 2 weeks later.

### 4.1 Improved hematological status

The mean 5.6% increase in tHb_mass_ for a hypoxic dose of 528 h confirmed the 1.0%–1.1%/100 h gain previously reported (Wehrlin et al., 2016), while the observed range (2.1%–11.0%) is consistent with the large inter-individual variability in the hematological response reported amongst swimmers of similar performance level exposed to the same environmental conditions and similar training plans (Friedmann et al., 2005; Wachsmuth et al., 2013; Bonne et al., 2014; Astridge et al., 2024). The present results confirm the well-described substantial inter- and intra-individual variations in the altitude-induced changes in tHb_mass_ in elite endurance athletes (Hauser et al., 2017; Skattebo and Hallén, 2022), leading to conclude that so-called responders and non-responders are not a “fixed trait.” Many causes have been put forward to explain such variability in elite athletes (Mujika et al., 2019), such as iron status (Govus et al., 2015), fatigue (Schmitt et al., 2016), health and nutritional status (Skattebo and Hallén, 2022), or sleep alteration (Van Cutsem and Pattyn, 2022). In this study, only the changes in serum iron correlated with tHb_mass_ increase. Although the present results did not confirm the major role of pre-altitude ferritin level on tHb_mass_ adaptation (maybe due to the combination of male and female swimmers inducing a large inter-individual variability), iron metabolism seemed paramount for inducing a significant erythropoiesis, confirmed by the observed increases in red blood cell count, hemoglobin concentration and hematocrit, along with declines of ∼30% and ∼15% in mean serum iron and mean ferritin values, respectively (Table 1).

The observed mean increase in hemoglobin concentration (14.4–15.6 g/dL) and hematocrit (42.8%–47.3%) is in keeping with changes (14.2–15.3 g/dL and 42.8%–47.5%, respectively) reported in a recent investigation on national and international caliber cross-country skiers during 4 weeks of normobaric “live-high, train-low and high” at a simulated altitude of 2,250–2,500 m. However, these hematological adaptations, and the reported 4.2% increase in tHb_mass_ did not bring about a short-term development of maximal endurance performance and maximal oxygen uptake (Kettunen et al., 2023). Skattebo and Hallén (Skattebo and Hallén, 2022) also studied elite endurance athletes over several 3-week altitude sojourns (∼2,000 m): in keeping with the present study, they observed significant increments in tHb_mass_ and hemoglobin concentration, but not in hematocrit. The latter observation could have been due to the high pre-altitude hematocrit of the participants (46.0%). Also in contrast with the present study, mean corpuscular hemoglobin concentration increased with altitude training (Skattebo and Hallén, 2022). This difference could have been related with the small reduction in training volume in the days prior to the end of the camp (Figure 3A). In this sense, small declines in mean corpuscular hemoglobin and mean corpuscular hemoglobin concentration have previously been observed in highly trained swimmers during a taper phase (Mujika et al., 1997). The potential clinical significance of such changes, however, remains to be elucidated. Whether the observed hematological changes could have been affected by technical variability and/or dehydration-induced hemoconcentration also remains to be elucidated. In this respect, USG values were generally consistent with an adequate hydration status throughout the camp, but it is worth mentioning that two of the participants presented USG values of 1.0208 and 1.0259, indicative of minimal and significant dehydration, respectively (Casa et al., 2000).

### 4.2 Swimming performance

The performance improvement observed during the standardized swimming set on days 3, 10 and 17 of the altitude training camp should be put into perspective, considering the usual performance decline upon athletes’ arrival at altitude. This improvement indicates that the hematological adaptations observed during such an altitude camp, suggestive of an improved blood oxygen carrying capacity, along with improved body composition, seemed to be beneficial for swim performance at altitude. On the other hand, enhanced altitude performance cannot be directly linked to competition performance at sea level. Indeed, several studies failed to observe a direct link between altitude-induced hematological benefits and performance benefits in subsequent sea level competition (Rodríguez et al., 2015; Wachsmuth et al., 2013; Bonne et al., 2014; Gough et al., 2012; Robertson et al., 2010). Recently, Mujika et al. (2023) did not find significant differences in tHb_mass_ among swimmers specializing in events ranging from 200 m to 10 km (open water), and swimmers’ best performances were not achieved in time with peak tHb_mass_ values. Similarly, and in keeping with the present results, Astridge et al. (2024) reported a lack of performance changes either immediately after or 3 weeks after a 3-week altitude camp at 2,320 m, despite enhanced tHb_mass_ values. Taken together, the above results reinforce the idea that although hematological benefits obtained during altitude camps can contribute to the quality of training *during* the camp itself, other factors may be stronger determinants of subsequent sea level swimming performance (Wachsmuth et al., 2013; Mujika et al., 2023). For instance, the optimal timing for performance peaking upon return to sea level after an altitude training camp, which itself is probably influenced by factors such as an athlete’s time course of hematological and non-hematological altitude de-acclimatization, and/or the individual response to the pre-competition taper program (Mujika et al., 2019).

### 4.3 Daily assessments and skinfolds

Mujika et al. (2019) highlighted the importance of an effective monitoring system to assess fitness and fatigue responses to altitude training, maximize the potential benefits of altitude training and limit the risk of illness, maladaptation or overtraining. The monitoring system implemented in this investigation included all five of the monitoring tools considered “essential” by the above authors (i.e., training intensity, training volume, pre-altitude iron status, questionnaires on athletes’ wellness, fatigue and recovery, and body mass), all four of the “important” tools (i.e., tHb_mass_, resting heart rate and SpO_2_, body composition), and one “optional” tool (i.e., USG). As can be observed in Figure 3, most of these markers remained relatively stable throughout the camp. The “stability” of the markers (despite small fluctuations possibly related with changes in training load and the presence or absence of early morning dryland training sessions) could probably be attributed to the participants’ extensive previous altitude training experience on the one hand, and the individualized adjustments to the training program implemented on the basis of the daily monitoring data.

Body mass (Figure 3D) and skinfold thickness (Figure 2D) declined throughout the altitude sojourn. These decrements could be beneficial to performance in the lead-up to a major competition, and are in line with previously reported modifications in body composition in swimmers performing altitude training camps (Bonne et al., 2014; Saw et al., 2018). This observation, along with the lack of negative outcomes in the other reported markers of athlete adaptation, underlines the practical value of daily assessments and multi-faceted approach in elite athletes performing training camps at altitude (Mujika et al., 2019; Saw et al., 2018).

### 4.4 Limitations

The lack of a control group and the small participant numbers should be considered as the main limitations of this study, as they pose a threat to the internal validity, generalizability and causality of the findings. In addition, there was no direct comparison of swimming performance measured before vs. after the altitude camp, only a comparison with the of the athletes’ personal best times; such design precludes differentiating the effects of altitude *per se* and the effects induced by the training program. However, engaging a formal control group at sea level is usually not an option when working with a small group of elite athletes. Notwithstanding these limitations, the present results allow initial exploration and provide baseline data for future research and practical purposes.

### 4.5 Practical applications

Strategically planned altitude training camps a few weeks ahead of major competitions is likely beneficial for swimmers’ hematological status and some sea level performance-determining factors in elite swimmers. Nevertheless, the performance enhancement that usually takes place at altitude appears to be multi-factorial and not only mediated by the erythropoietic response (Schmitt et al., 2016), and whether altitude-induced gains are converted to sea level swimming performance gains depends on multiple factors. Altogether, the present results underscore the relevance of adequate planning, training, and monitoring potential confounding factors (e.g., fatigue, iron status, nutrition, hydration, sleep) to maximize the potential benefits of altitude training camps.

## 5 Conclusion

An altitude training camp of 22 days at 2,320 m was beneficial for altitude performance, tHb_mass_ and the hematological status of elite swimmers. These changes, however, did not clearly translate into improved sea level competition performance either immediately or 2 weeks after the camp. The present study confirms the large inter-subject variability in hematological responses to altitude training; and that the improvement in performance at altitude and sea level may depend on factors other than the increase in tHb_mass_ alone.

## Data Availability

The raw data supporting the conclusions of this article will be made available by the authors, without undue reservation.
